# EXPLORING OPPORTUNITIES TO FOSTER GERONTOLOGY EDUCATION AND GLOBAL ENGAGEMENT

**DOI:** 10.1093/geroni/igae098.0050

**Published:** 2024-12-31

**Authors:** Ramraj Gautam, Linda Hollis, Jennifer Mendez

**Affiliations:** University of Massachusetts Lowell, Lowell, Massachusetts, United States; University of Arizona, Tucson, Arizona, United States; Wayne State University School of Medicine, Detroit, Michigan, United States

## Abstract

The Academy for Gerontology in Higher Education (AGHE) helps advance gerontology, geriatrics, and age-friendly instruction through the development of curriculum, educational, and training materials. Much of this work is done in the context of the United States. GSA/AGHE being an international organization there remains a further need to bring global gerontology educators and experts together. Using the UN Decade of Healthy Aging framework, the purpose of this study was a) to learn more about international gerontology education programs in order to collaborate and share materials, and b) to explore opportunities in increasing international aging programs. The study protocol was IRB-approved. International gerontology educators were recruited through announcements in aging newsletters, GSA Connect, interest groups, AGHExchange, and through GSA 2023 international presenters’ listserv. Twenty-six participants representing academic institutions from ten different countries responded to the Qualtrics survey. Several strategies were suggested for sharing gerontology education resources: curricula and syllabi sharing through cloud servers, inviting international guest lectures, involving students in international and intergenerational projects, and collaborations with international educators. Among the four World Health Organization decades of healthy aging 2022-23 focus areas, participants ranked Age-Friendly Environments as number one, followed by combating ageism, long-term care, and integrated care. 61.5% of respondents were not a member of AGHE – GSA and 85.7% of participants showed interest in joining. Plans to incorporate the suggested strategies and future collaboration international gerontology educator opportunities will be discussed.

